# A model of blood flow in the mesenteric arterial system

**DOI:** 10.1186/1475-925X-6-17

**Published:** 2007-05-08

**Authors:** Thusitha DS Mabotuwana, Leo K Cheng, Andrew J Pullan

**Affiliations:** 1 Bioengineering Institute, The University of Auckland, Private Bad 92019, Auckland 1142, New Zealand; 2 Department of Engineering Science, The University of Auckland, Private Bag 92019, Auckland 1142, New Zealand

## Abstract

**Background:**

There are some early clinical indicators of cardiac ischemia, most notably a change in a person's electrocardiogram. Less well understood, but potentially just as dangerous, is ischemia that develops in the gastrointestinal system. Such ischemia is difficult to diagnose without angiography (an invasive and time-consuming procedure) mainly due to the highly unspecific nature of the disease.

Understanding how perfusion is affected during ischemic conditions can be a useful clinical tool which can help clinicians during the diagnosis process. As a first step towards this final goal, a computational model of the gastrointestinal system has been developed and used to simulate realistic blood flow during normal conditions.

**Methods:**

An anatomically and biophysically based model of the major mesenteric arteries has been developed to be used to simulate normal blood flows. The computational mesh used for the simulations has been generated using data from the Visible Human project. The 3D Navier-Stokes equations that govern flow within this mesh have been simplified to an efficient 1D scheme. This scheme, together with a constitutive pressure-radius relationship, has been solved numerically for pressure, vessel radius and velocity for the entire mesenteric arterial network.

**Results:**

The computational model developed shows close agreement with physiologically realistic geometries other researchers have recorded *in vivo*. Using this model as a framework, results were analyzed for the four distinct phases of the cardiac cycle – diastole, isovolumic contraction, ejection and isovolumic relaxation. Profiles showing the temporally varying pressure and velocity for a periodic input varying between 10.2 kPa (77 mmHg) and 14.6 kPa (110 mmHg) at the abdominal aorta are presented. An analytical solution has been developed to model blood flow in tapering vessels and when compared with the numerical solution, showed excellent agreement.

**Conclusion:**

An anatomically and physiologically realistic computational model of the major mesenteric arteries has been developed for the gastrointestinal system. Using this model, blood flow has been simulated which show physiologically realistic flow profiles.

## Background

The purpose of our current research is to develop an extensible anatomically and biophysically based computational model of the mesenteric arterial system, which is the main blood supply to the human intestine, and to use this model to carefully examine intestinal blood flow. We believe that such a model could have clinical applications particularly with relation to mesenteric ischemia, a complex vascular problem that arises due to a narrowing or blockage of blood vessels that supply oxygenated blood to the small and large intestines, for which accurate diagnosis is often delayed. The prevalence of mesenteric ischemia is increasing worldwide as the population ages and represents one of the most threatening abdominal conditions in elderly patients [1]. The delayed diagnosis results in an estimated mortality rate of around 60 – 80% [2,3] and is usually attributed to the unspecific nature of the abdominal "gut pain". It is difficult, even for the trained specialist, in discriminating ischemia from the many other types of gut pains (which are more common and less severe). Due to the lack of any non-invasive clinical indicators which can be used to determine the viability of the intestinal smooth muscle before any irreversible changes have occurred [4], very little is known about the development and progression of gastrointestinal ischemia. The computational framework described below allows the effect of a number of different scenarios to be explored – something not possible when dealing with patients. It also allows an establishment of a database of normal range of mesenteric circulation that can be used to investigate deviations from normality. This could help in the early diagnosis of mesenteric ischemia in order to prevent secondary diseases such as ischemic colitis, gangrene and perforation of the bowel. Such a database would allow comparison of a subject's pathological profiles with those from a healthy subject and an appreciation for various model parameters can help identify the pathologic conditions (such as how stiff or compliant the arteries are) involved. Further, numerical simulations could be used as a tool when using shape optimization theory in the development of prosthetic devices or vascular grafts, designing new prototypes, providing specific design indications for the realization of various surgical procedures and developing training beds for new vascular surgeons [5].

Since the introduction of the one-dimensional modeling of the human arterial system by Euler in 1775 [6], many blood flow models have evolved, but a single model which can fully capture all aspects of the hemodynamics of the human arterial system is yet to be developed. Arguably, this can be attributed to the non-linear nature of blood flow in a very complex, mostly viscoelastic vascular network full of non-planer, tapering branches. To make physiologically realistic analyses of the cardiovascular system even harder and more complex, the vascular system can simply regulate itself – arterioles can contract and pulse rate can increase when blood pressure drops, while an increase in blood pressure can result in a dilation of the arterioles (hence a reduction of the periphery resistance to flow) and therefore a lower heart rate [5]. Even the blood, which consists of 55% plasma and 45% cells (erythrocytes, leukocytes and platelets), is quite a complex substance on its own showing many anomalous properties when compared to a typical fluid. The presence of backup systems (e.g., vascular loops seen mainly in the mesenteric vasculature) further adds complexity to realistic blood flow modeling.

Much of the literature on hemodynamics is still confined to either simple networks or idealized geometry (e.g., symmetry in the sagittal plane, identical daughter vessels at bifurcations, planar geometry, straight vessels with no tapering and rigid walled approximations [7,8]). However, some studies have investigated blood flow patterns using anatomically realistic geometries. Several imaging modalities (including Magnetic Resonance (MR) imaging [9-11], variations of Computed Tomography (CT) imaging [12,13], reconstruction from biplane angiography with intravascular ultrasound [14,15] and MR Angiography (MRA) [16,17]) have been used to create such geometry for various sections of the human arterial system, most commonly the coronary arteries [14,15,18,19], femoral arteries [20,21], carotid bifurcation [22,23] and the aorto-iliac bifurcation [10,16], but to the authors' knowledge there have been no efforts in the past to reconstruct the mesenteric arteries.

A few three-dimensional models have been developed in recent years to study the effects of wall shear stresses on the development of lesions and atherosclerosis in simple arterial networks [8,24]. However, solving a full scale three-dimensional computational fluid dynamics (CFD) algorithm on a complex network is currently not feasible; firstly due to the lack of a large set of morphological data and secondly because it is computationally prohibitive. Therefore, in this paper we treat the blood flow within the mesenteric system as one-dimensional and solve this model using numerical techniques developed previously by Smith et al [18]. This provides an efficient numerical scheme to model pulsatile three-dimensional blood flow using a single dimension, and simulate vessel diameter changes and pressure distributions.

## Methods

This section details the data digitization process, finite element creation, model development and the governing blood flow equations. Numerical analysis and stability issues are also discussed. All model creation and numerical results and visualization were generated using the custom developed software package known as CMISS (  ).

### Data digitization

**Figure 1:**
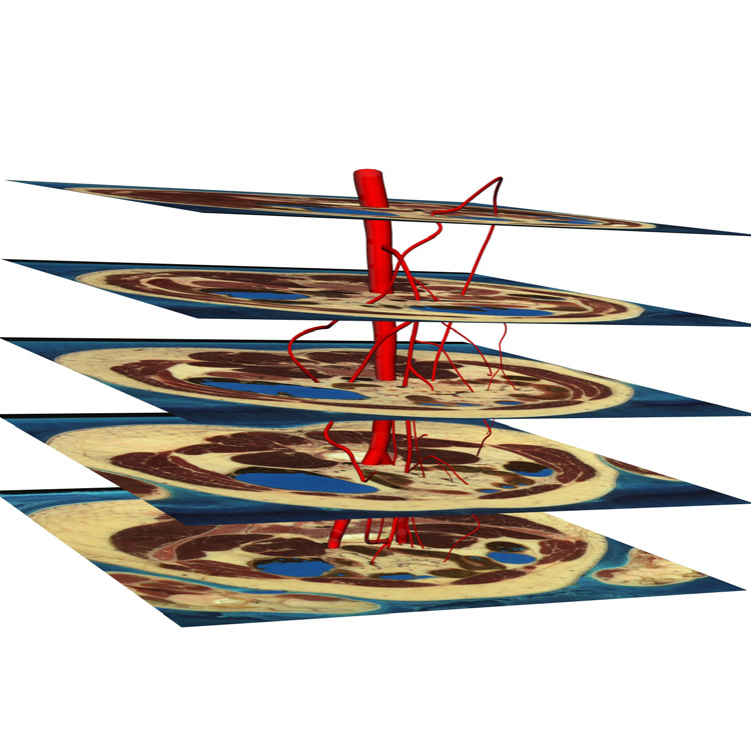
Anterior view of a subset of five images from the Visible Human dataset showing how the mesenteric arteries were created.

Our computational mesh was created using the high resolution (0.3 mm/pixel) male Visible Human (VH) dataset which contains 2D axial slices, each 1 mm apart. The centre-line of the mesenteric arteries with a radius of approximately 0.5 mm and greater was visually identified and traced (to give a total of 898 raw data points) on a vertical segment of 251 mm of the human body. By stacking these images as shown in Fig. 1, an initial 3D model was constructed. The abdominal aorta, Superior Mesenteric Artery (SMA), Inferior Mesenteric Artery (IMA), common iliac arteries and the middle colic artery were relatively easy to trace on the VH images, but the actual vessel boundaries of the branches of the SMA were difficult to determine, and anatomical texts [25] were used to augment the digitized data.

### Finite element model

**Figure 2:**
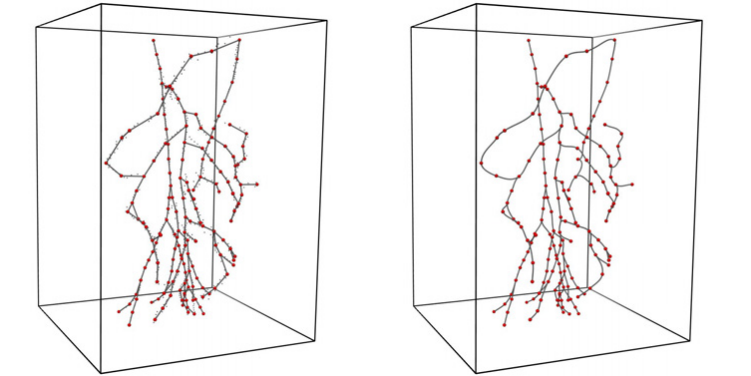
Finite element creation and fitting of mesenteric arteries. (a) Traced data points (smaller black spheres), node selection (larger red spheres) and linear element creation. (b) Fitted mesenteric artery network with nodes (red spheres).

A total of 188 points were selected at regular intervals from the set of 898 raw data points obtained after digitization and used as nodes (red spheres in Fig. 2(a)) in the construction of the finite element mesh. The selected nodes were then connected linearly to form the initial, linear finite element model. The linear elements were then fitted to the entire digitized dataset using a 1D cubic Hermite interpolation scheme (refer to [26] for details on geometric fitting using cubic Hermite elements). The final resulting mesh of this fitting process is the smooth network shown in Fig. 2(b) consisting of a total of 159 vessel segments with 25 bifurcations. Within the cubic Hermite mesh, a total of 834 points were placed in the local finite element space such that there was an average grid point spacing of 1.3 mm. These points were used as the finite difference solution points in our blood flow calculations (see Section "Modeling Blood Flow").

### Initial radius assignment

**Figure 3:**
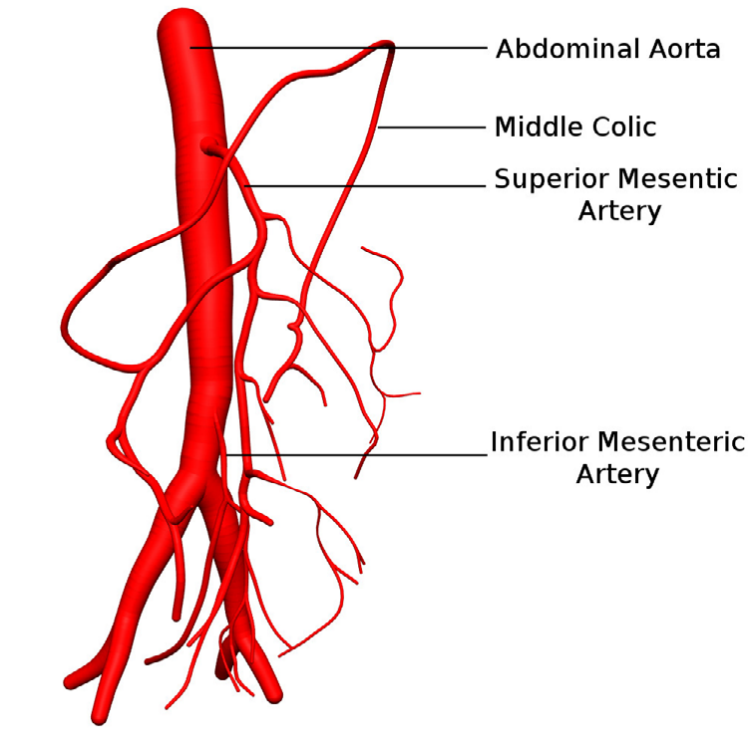
Anterior view of the 3D anatomical model of a segment of abdominal aorta with the superior and inferior mesenteric arteries and their sub-divisional branches, with assigned radii.

The initial unstressed arterial radius (defined as the radius at 0 kPa pressure) at each node shown in Fig. 2(a) was determined from the VH images. Where possible, these radii were validated against other published data to ensure their accuracy, and the values of the abdominal aorta, SMA and IMA were in good comparison with published material (see Table 1). The radii values assigned at the nodes were then interpolated linearly to create the geometry shown in Fig. 3.

**Table 1:** Comparison of initial radii used for abdominal aorta, SMA and IMA.

Reference	Radius (mm)		
	
	Abdominal Aorta	SMA	IMA
Olufsen et al [9]	8.5	3.3	2
Lee et al [39]	-	4*	3*
Peifer et al [11]	-	3.85	3
Current model	7.5	4.2	3.4

Shown in Fig. 3 is the model constructed after the initial radii were assigned. *Original diameter values have been converted to radius values.

### Modeling blood flow

#### Governing flow equations

Several approaches have been used in the literature to model blood flow in large vessels in the cardiovascular system. Modeling the pulsatile flow using Fourier analysis [27,28] and using the mass and momentum conservation equations coupled with a state equation [6,9,18] seem to be two of the most widely used approaches, while several other techniques, including flow modeling using closed-loop systems mimicking electrical circuits [29,30], can also be found. Most physiological parameters (including the temporal variations in the cardiac cycle itself) are more directly applicable to time domain models and we believe that time domain analysis (as opposed to frequency domain analysis) would provide additional, easier-to-interpret information in terms of subject pressure and flow profiling, especially where genesis and progression of ischemic conditions would be studied. On a secondary note, clinicians and most non-technical personnel normally find it difficult to interpret results explained in the frequency domain and would prefer the more intuitive time-domain approach.

In this study, blood is assumed to be Newtonian fluid a common assumption in blood flow analysis in large to medium sized vessels [5,9,16]. A typical Reynolds number in the abdominal aorta is around 590 [16] and this is well below the critical Reynolds number (which is generally considered to be 2300) above which the transition from laminar to turbulent flow usually occurs [31], therefore laminar flow is assumed throughout the study. Further, blood is considered to be an incompressible, homogeneous fluid with an axisymmetric flow and constant viscosity. Under these assumptions, using a cylindrical coordinate system (*r, θ, x*) where the *x *axis is aligned with the local vessel axial direction and assuming a zero velocity in the circumferential direction, the complete 3-dimensional Navier-Stokes equations can be reduced to a set of 1-dimensional flow equations:

∂R∂t+V∂R∂x+R2∂V∂x=0
 MathType@MTEF@5@5@+=feaafiart1ev1aaatCvAUfKttLearuWrP9MDH5MBPbIqV92AaeXatLxBI9gBaebbnrfifHhDYfgasaacH8akY=wiFfYdH8Gipec8Eeeu0xXdbba9frFj0=OqFfea0dXdd9vqai=hGuQ8kuc9pgc9s8qqaq=dirpe0xb9q8qiLsFr0=vr0=vr0dc8meaabaqaciaacaGaaeqabaqabeGadaaakeaadaWcaaqaaiabgkGi2kabdkfasbqaaiabgkGi2kabdsha0baacqGHRaWkcqWGwbGvdaWcaaqaaiabgkGi2kabdkfasbqaaiabgkGi2kabdIha4baacqGHRaWkdaWcaaqaaiabdkfasbqaaiabikdaYaaadaWcaaqaaiabgkGi2kabdAfawbqaaiabgkGi2kabdIha4baacqGH9aqpcqaIWaamaaa@444E@

and

∂V∂t+2(1−α)VR∂R∂t+αV∂V∂x+1ρ∂p∂x=2υR[∂vx∂r]R
 MathType@MTEF@5@5@+=feaafiart1ev1aaatCvAUfKttLearuWrP9MDH5MBPbIqV92AaeXatLxBI9gBaebbnrfifHhDYfgasaacH8akY=wiFfYdH8Gipec8Eeeu0xXdbba9frFj0=OqFfea0dXdd9vqai=hGuQ8kuc9pgc9s8qqaq=dirpe0xb9q8qiLsFr0=vr0=vr0dc8meaabaqaciaacaGaaeqabaqabeGadaaakeaadaWcaaqaaiabgkGi2kabdAfawbqaaiabgkGi2kabdsha0baacqGHRaWkcqaIYaGmcqGGOaakcqaIXaqmcqGHsisliiGacqWFXoqycqGGPaqkdaWcaaqaaiabdAfawbqaaiabdkfasbaadaWcaaqaaGGaaiab+jGi2kabdkfasbqaaiabgkGi2kabdsha0baacqGHRaWkcqWFXoqycqWGwbGvdaWcaaqaaiabgkGi2kabdAfawbqaaiabgkGi2kabdIha4baacqGHRaWkdaWcaaqaaiabigdaXaqaaiab=f8aYbaadaWcaaqaaiabgkGi2kabdchaWbqaaiabgkGi2kabdIha4baacqGH9aqpdaWcaaqaaiabikdaYiab=v8a1bqaaiabdkfasbaadaWadaqaamaalaaabaGaeyOaIyRaemODay3aaSbaaSqaaiabdIha4bqabaaakeaacqGHciITcqWGYbGCaaaacaGLBbGaayzxaaWaaSbaaSqaaiabdkfasbqabaaaaa@636A@

where *p, R, V, ρ *and *ν *represent pressure, inner vessel radius, average velocity, blood density and blood viscosity respectively. The parameter α is used to specify the shape of the axial velocity profile, with α = 1 corresponding to a flat profile.

The right hand side of (2) can be determined by specifying an axial velocity profile in the *x *direction (*v*_*x*_) of the form

vx=α2−αV[1−(rR)2−αα−1]
 MathType@MTEF@5@5@+=feaafiart1ev1aaatCvAUfKttLearuWrP9MDH5MBPbIqV92AaeXatLxBI9gBaebbnrfifHhDYfgasaacH8akY=wiFfYdH8Gipec8Eeeu0xXdbba9frFj0=OqFfea0dXdd9vqai=hGuQ8kuc9pgc9s8qqaq=dirpe0xb9q8qiLsFr0=vr0=vr0dc8meaabaqaciaacaGaaeqabaqabeGadaaakeaacqWG2bGDdaWgaaWcbaGaemiEaGhabeaakiabg2da9maalaaabaacciGae8xSdegabaGaeGOmaiJaeyOeI0Iae8xSdegaaiabdAfawnaadmaabaGaeGymaeJaeyOeI0YaaeWaaeaadaWcaaqaaiabdkhaYbqaaiabdkfasbaaaiaawIcacaGLPaaadaahaaWcbeqaamaalaaabaGaeGOmaiJaeyOeI0Iae8xSdegabaGae8xSdeMaeyOeI0IaeGymaedaaaaaaOGaay5waiaaw2faaaaa@4673@

**Figure 4:**
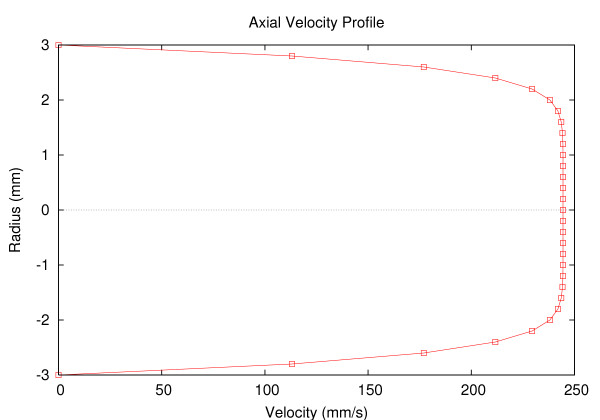
Axial velocity flow profile across the vessel with radius of 3 mm and α = 1.1.

This form in (3) was deemed suitable by Hunter [32] to give a compromise fit to experimental data obtained at various different points in the cardiac cycle. The form of the axial velocity profile with a value of α = 1.1, V = 200 mm/s and R = 3 mm is shown in Fig 4.

It should be noted that there are two singularities with this equation; when α = 1 and when R = 0. When α = 1 it is not physiological and results in a flow profile which is a step function with no flow at the walls and maximum flow just off the walls. The case where R = 0 corresponds to a fully collapsed or occluded vessel. Although rare, this is a condition that can occur physiologically and it can be represented in the model by decoupling a particular vessel segment and replacing the terminals with a no-flow boundary condition.

Further manipulation of equations (1) – (3) gives

∂V∂t+(2α−1)V∂V∂x+2(α−1)V2R∂R∂x+1ρ∂p∂x=−2ναα−1VR2
 MathType@MTEF@5@5@+=feaafiart1ev1aaatCvAUfKttLearuWrP9MDH5MBPbIqV92AaeXatLxBI9gBaebbnrfifHhDYfgasaacH8akY=wiFfYdH8Gipec8Eeeu0xXdbba9frFj0=OqFfea0dXdd9vqai=hGuQ8kuc9pgc9s8qqaq=dirpe0xb9q8qiLsFr0=vr0=vr0dc8meaabaqaciaacaGaaeqabaqabeGadaaakeaadaWcaaqaaiabgkGi2kabdAfawbqaaiabgkGi2kabdsha0baacqGHRaWkcqGGOaakcqaIYaGmiiGacqWFXoqycqGHsislcqaIXaqmcqGGPaqkcqWGwbGvdaWcaaqaaiabgkGi2kabdAfawbqaaiabgkGi2kabdIha4baacqGHRaWkcqaIYaGmcqGGOaakcqWFXoqycqGHsislcqaIXaqmcqGGPaqkdaWcaaqaaiabdAfawnaaCaaaleqabaGaeGOmaidaaaGcbaGaemOuaifaamaalaaabaGaeyOaIyRaemOuaifabaGaeyOaIyRaemiEaGhaaiabgUcaRmaalaaabaGaeGymaedabaGae8xWdihaamaalaaabaGaeyOaIyRaemiCaahabaGaeyOaIyRaemiEaGhaaiabg2da9iabgkHiTiabikdaYmaalaaabaGae8xVd4Mae8xSdegabaGae8xSdeMaeyOeI0IaeGymaedaamaalaaabaGaemOvayfabaGaemOuai1aaWbaaSqabeaacqaIYaGmaaaaaaaa@66B5@

Equations (1) and (4) provide us with two equations for the three unknowns, *P*, *R *and *V*. A third equation can now be determined by taking the vessel mechanics into account, and in this study we have chosen a pressure-radius relationship of the form

p(R)=G0[(RR0)β−1]
 MathType@MTEF@5@5@+=feaafiart1ev1aaatCvAUfKttLearuWrP9MDH5MBPbIqV92AaeXatLxBI9gBaebbnrfifHhDYfgasaacH8akY=wiFfYdH8Gipec8Eeeu0xXdbba9frFj0=OqFfea0dXdd9vqai=hGuQ8kuc9pgc9s8qqaq=dirpe0xb9q8qiLsFr0=vr0=vr0dc8meaabaqaciaacaGaaeqabaqabeGadaaakeaacqWGWbaCcqGGOaakcqWGsbGucqGGPaqkcqGH9aqpcqWGhbWrdaWgaaWcbaGaeGimaadabeaakmaadmaabaWaaeWaaeaadaWcaaqaaiabdkfasbqaaiabdkfasnaaBaaaleaacqaIWaamaeqaaaaaaOGaayjkaiaawMcaamaaCaaaleqabaacciGae8NSdigaaOGaeyOeI0IaeGymaedacaGLBbGaayzxaaaaaa@3EFA@

where G_0 _and *β *are constants defining a particular wall behavior and R_0 _is the initial unstressed vessel radius. This was the empirical relationship originally deduced by Hunter [32] and then adapted by Smith et al [18] in their work. The chosen form assumes a purely elastic behavior of the arterial wall and closely agrees with the conclusions drawn by Saito et al [33] who concluded from their experiments that in large artery models, viscoelasticity may be neglected and the arterial walls may be considered purely elastic. Similar pressure-radius relationships have been proposed by Sherwin et al [6] and Olufsen et al [9] by assuming a purely elastic wall.

#### Flow in a single vessel

The governing equations cannot be solved analytically and the use of numerical techniques is required. The Two-Step-Lax-Wendroff finite difference method was selected as a suitable explicit scheme as it is second order accurate in both space and time while eliminating large numerical dissipations [18,32].

Equations (1), (4) and (5) were then solved numerically using the above finite differencing technique for an N grid point arterial segment to determine the values (*P*, *R *and *V*) at each of the interior grid points (*i *= 2 to N-1, where *i *denotes a grid point) while a boundary scheme is required to determine the values at the two ends of the vessel segment. The specified boundary condition was chosen to be pressure, as opposed to velocity or flow pulses chosen by Parker et al [34] in their work, as pressure can be measured in a clinical environment and is also less sensitive to small measurement errors. Radius is simply a function of pressure via the constitutive equation (5) and by considering the characteristic directions along which information propagates in (*x,t*) space, an expression for velocity at the first and last grid points (*i *= 1 and *i *= N respectively) in a single vessel can be derived (refer to [18] for details).

Following the studies of [18,32] G_0 _was set to 21.2 kPa (158 mmHg) and β was set to 2.0 due to the nature of the arterial walls. A value of α = 1.1 was chosen to define the axial velocity profile. Blood density was assumed to be 1.05 gcm^-3 ^and viscosity was considered to be 3.2 cm^2^s^-1 ^(these parameter values are used for all simulations presented here).

#### Analytical test solution

**Figure 5:**
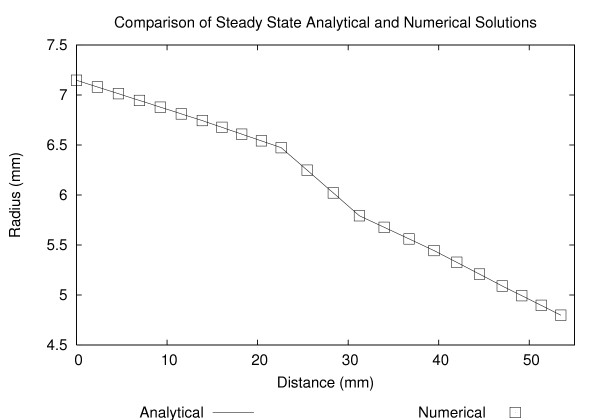
Steady-state analytical and numerical solutions for a tapering segment of the descending abdominal aorta when the inlet pressure is raised from 12.6 kPa to 14.6 kPa over 0.2 s.

In order to test our numerical scheme and its implementation, we simulated the flow in an approximately 55 mm long tapering section of the abdominal aorta (the chosen location was just below the SMA and slightly above the IMA since we needed a single vessel with no branching) and the initial conditions were set to pi0
 MathType@MTEF@5@5@+=feaafiart1ev1aaatCvAUfKttLearuWrP9MDH5MBPbIqV92AaeXatLxBI9gBaebbnrfifHhDYfgasaacH8akY=wiFfYdH8Gipec8Eeeu0xXdbba9frFj0=OqFfea0dXdd9vqai=hGuQ8kuc9pgc9s8qqaq=dirpe0xb9q8qiLsFr0=vr0=vr0dc8meaabaqaciaacaGaaeqabaqabeGadaaakeaacqWGWbaCdaqhaaWcbaGaemyAaKgabaGaeGimaadaaaaa@308B@ = 12.5 kPa, Ri0=R0i
 MathType@MTEF@5@5@+=feaafiart1ev1aaatCvAUfKttLearuWrP9MDH5MBPbIqV92AaeXatLxBI9gBaebbnrfifHhDYfgasaacH8akY=wiFfYdH8Gipec8Eeeu0xXdbba9frFj0=OqFfea0dXdd9vqai=hGuQ8kuc9pgc9s8qqaq=dirpe0xb9q8qiLsFr0=vr0=vr0dc8meaabaqaciaacaGaaeqabaqabeGadaaakeaacqWGsbGudaqhaaWcbaGaemyAaKgabaGaeGimaadaaOGaeyypa0JaemOuai1aaSbaaSqaaiabicdaWmaaBaaameaacqWGPbqAaeqaaaWcbeaaaaa@3539@ and Vi0=0
 MathType@MTEF@5@5@+=feaafiart1ev1aaatCvAUfKttLearuWrP9MDH5MBPbIqV92AaeXatLxBI9gBaebbnrfifHhDYfgasaacH8akY=wiFfYdH8Gipec8Eeeu0xXdbba9frFj0=OqFfea0dXdd9vqai=hGuQ8kuc9pgc9s8qqaq=dirpe0xb9q8qiLsFr0=vr0=vr0dc8meaabaqaciaacaGaaeqabaqabeGadaaakeaacqWGwbGvdaqhaaWcbaGaemyAaKgabaGaeGimaadaaOGaeyypa0JaeGimaadaaa@3255@ for each grid point *i*. The initial radius was specified at various locations along the vessel (using the information extracted during the digitizing process) and the variation in radius along each segment between 2 specified locations was assumed to be linear. The pressure at the inlet was raised from 12.6 kPa to 14.6 kPa over 0.2 s (the approximate pressure change in the heart during the ejection phase shown in Fig. 7) and the spatial changes were plotted in Fig. 5.

**Figure 6:**
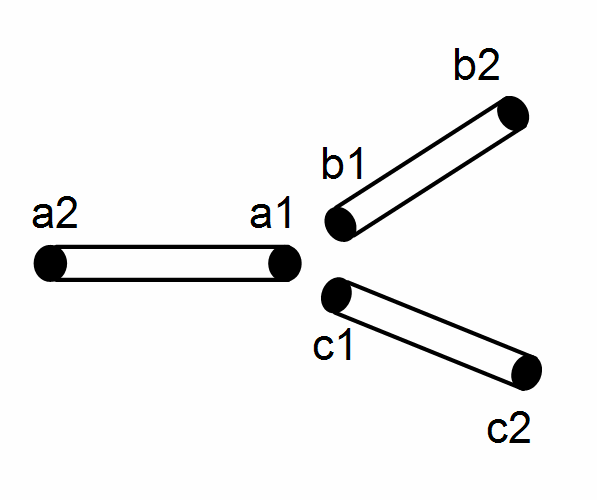
Grid points at a bifurcation.

**Figure 7:**
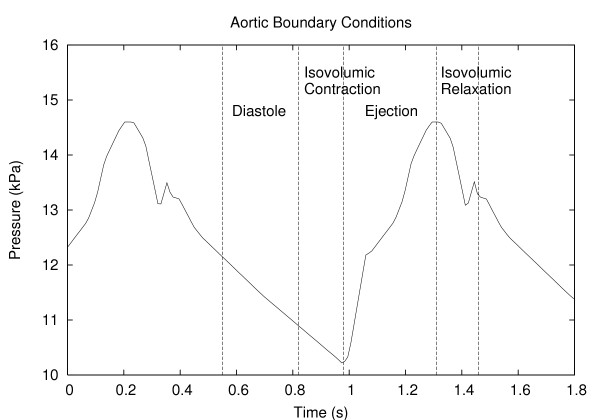
Aortic inflow pressure boundary condition.

To validate the above results, we also derived a steady state analytical solution using mass conservation. Considering the vessel area *S *= *πR*^*2*^, a steady state expression can be derived from (2) and (3) where all *∂/∂t *terms are set to 0.

αVdVdx+1ρ∂p∂x=−2πυαα−1VS
 MathType@MTEF@5@5@+=feaafiart1ev1aaatCvAUfKttLearuWrP9MDH5MBPbIqV92AaeXatLxBI9gBaebbnrfifHhDYfgasaacH8akY=wiFfYdH8Gipec8Eeeu0xXdbba9frFj0=OqFfea0dXdd9vqai=hGuQ8kuc9pgc9s8qqaq=dirpe0xb9q8qiLsFr0=vr0=vr0dc8meaabaqaciaacaGaaeqabaqabeGadaaakeaaiiGacqWFXoqycqWGwbGvdaWcaaqaaiabdsgaKjabdAfawbqaaiabdsgaKjabdIha4baacqGHRaWkdaWcaaqaaiabigdaXaqaaiab=f8aYbaadaWcaaqaaiabgkGi2kabdchaWbqaaiabgkGi2kabdIha4baacqGH9aqpcqGHsislcqaIYaGmcqWFapaCdaWcaaqaaiab=v8a1jab=f7aHbqaaiab=f7aHjabgkHiTiabigdaXaaadaWcaaqaaiabdAfawbqaaiabdofatbaaaaa@4C36@

Using a constant flow rate *Q = VS *and considering that initial radius *R*_0 _(hence *S*_0_) to be a function of distance *x*, equation (6) can be modified to:

[−αQ2S+G0β2ρS0.5β+1S00.5β]dSdx−G0β2ρS0−0.5β−1S0.5β+2dS0dx=−2πυαα−1Q
 MathType@MTEF@5@5@+=feaafiart1ev1aaatCvAUfKttLearuWrP9MDH5MBPbIqV92AaeXatLxBI9gBaebbnrfifHhDYfgasaacH8akY=wiFfYdH8Gipec8Eeeu0xXdbba9frFj0=OqFfea0dXdd9vqai=hGuQ8kuc9pgc9s8qqaq=dirpe0xb9q8qiLsFr0=vr0=vr0dc8meaabaqaciaacaGaaeqabaqabeGadaaakeaadaWadaqaaiabgkHiTmaalaaabaacciGae8xSdeMaemyuae1aaWbaaSqabeaacqaIYaGmaaaakeaacqWGtbWuaaGaey4kaSYaaSaaaeaacqWGhbWrdaWgaaWcbaGaeGimaadabeaakiab=j7aIbqaaiabikdaYiab=f8aYbaadaWcaaqaaiabdofatnaaCaaaleqabaGaeGimaaJaeiOla4IaeGynauJae8NSdiMaey4kaSIaeGymaedaaaGcbaGaem4uam1aa0baaSqaaiabicdaWaqaaiabicdaWiabc6caUiabiwda1iab=j7aIbaaaaaakiaawUfacaGLDbaadaWcaaqaaiabdsgaKjabdofatbqaaiabdsgaKjabdIha4baacqGHsisldaWcaaqaaiabdEeahnaaBaaaleaacqaIWaamaeqaaOGae8NSdigabaGaeGOmaiJae8xWdihaaiabdofatnaaDaaaleaacqaIWaamaeaacqGHsislcqaIWaamcqGGUaGlcqaI1aqncqWFYoGycqGHsislcqaIXaqmaaGccqWGtbWudaahaaWcbeqaaiabicdaWiabc6caUiabiwda1iab=j7aIjabgUcaRiabikdaYaaakmaalaaabaGaemizaqMaem4uam1aaSbaaSqaaiabicdaWaqabaaakeaacqWGKbazcqWG4baEaaGaeyypa0JaeyOeI0IaeGOmaiJae8hWda3aaSaaaeaacqWFfpqDcqWFXoqyaeaacqWFXoqycqGHsislcqaIXaqmaaGaemyuaefaaa@7BCA@

Now considering the variation of *R*_0 _to be linear with *x *between two grid points, i.e.,

*R*_0 _= *a *+ *bx *(8)

where *a, b *are constants that can be easily determined when radii at two locations along the vessel are known, and:

S0=πR02
 MathType@MTEF@5@5@+=feaafiart1ev1aaatCvAUfKttLearuWrP9MDH5MBPbIqV92AaeXatLxBI9gBaebbnrfifHhDYfgasaacH8akY=wiFfYdH8Gipec8Eeeu0xXdbba9frFj0=OqFfea0dXdd9vqai=hGuQ8kuc9pgc9s8qqaq=dirpe0xb9q8qiLsFr0=vr0=vr0dc8meaabaqaciaacaGaaeqabaqabeGadaaakeaacqWGtbWudaWgaaWcbaGaeGimaadabeaakiabg2da9GGaciab=b8aWjabdkfasnaaDaaaleaacqaIWaamaeaacqaIYaGmaaaaaa@3503@

we get

*S*_0 _= *π*(*a *+ *bx*)^2 ^(10)

Differentiating (10) with respect to *x *gives:

dS0dx=2π(a+bx)b
 MathType@MTEF@5@5@+=feaafiart1ev1aaatCvAUfKttLearuWrP9MDH5MBPbIqV92AaeXatLxBI9gBaebbnrfifHhDYfgasaacH8akY=wiFfYdH8Gipec8Eeeu0xXdbba9frFj0=OqFfea0dXdd9vqai=hGuQ8kuc9pgc9s8qqaq=dirpe0xb9q8qiLsFr0=vr0=vr0dc8meaabaqaciaacaGaaeqabaqabeGadaaakeaadaWcaaqaaiabdsgaKjabdofatnaaBaaaleaacqaIWaamaeqaaaGcbaGaemizaqMaemiEaGhaaiabg2da9iabikdaYGGaciab=b8aWnaabmaabaGaemyyaeMaey4kaSIaemOyaiMaemiEaGhacaGLOaGaayzkaaGaemOyaigaaa@3EAF@

and (8) and (11) can now be substituted into (7) to derive an expression for the variation of *S *with *x *for a single vessel segment:

dSdx=πG0βρb[π(a+bx)2]−0.5β−1S0.5β+2(a+bx)−2πναα−1Q−αQ2S2+G0β2ρS0.5β+2[π(a+bx)2]0.5β
 MathType@MTEF@5@5@+=feaafiart1ev1aaatCvAUfKttLearuWrP9MDH5MBPbIqV92AaeXatLxBI9gBaebbnrfifHhDYfgasaacH8akY=wiFfYdH8Gipec8Eeeu0xXdbba9frFj0=OqFfea0dXdd9vqai=hGuQ8kuc9pgc9s8qqaq=dirpe0xb9q8qiLsFr0=vr0=vr0dc8meaabaqaciaacaGaaeqabaqabeGadaaakeaadaWcaaqaaiabdsgaKjabdofatbqaaiabdsgaKjabdIha4baacqGH9aqpdaWcaaqaaGGaciab=b8aWnaalaaabaGaem4raC0aaSbaaSqaaiabicdaWaqabaGccqWFYoGyaeaacqWFbpGCaaGaemOyai2aamWaaeaacqWFapaCdaqadaqaaiabdggaHjabgUcaRiabdkgaIjabdIha4bGaayjkaiaawMcaamaaCaaaleqabaGaeGOmaidaaaGccaGLBbGaayzxaaWaaWbaaSqabeaacqGHsislcqaIWaamcqGGUaGlcqaI1aqncqWFYoGycqGHsislcqaIXaqmaaGccqWGtbWudaahaaWcbeqaaiabicdaWiabc6caUiabiwda1iab=j7aIjabgUcaRiabikdaYaaakmaabmaabaGaemyyaeMaey4kaSIaemOyaiMaemiEaGhacaGLOaGaayzkaaGaeyOeI0IaeGOmaiJae8hWda3aaSaaaeaacqWF9oGBcqWFXoqyaeaacqWFXoqycqGHsislcqaIXaqmaaGaemyuaefabaGaeyOeI0YaaSaaaeaacqWFXoqycqWGrbqudaahaaWcbeqaaiabikdaYaaaaOqaaiabdofatnaaCaaaleqabaGaeGOmaidaaaaakiabgUcaRmaalaaabaGaem4raC0aaSbaaSqaaiabicdaWaqabaGccqWFYoGyaeaacqaIYaGmcqWFbpGCaaWaaSaaaeaacqWGtbWudaahaaWcbeqaaiabicdaWiabc6caUiabiwda1iab=j7aIjabgUcaRiabikdaYaaaaOqaamaadmaabaGae8hWda3aaeWaaeaacqWGHbqycqGHRaWkcqWGIbGycqWG4baEaiaawIcacaGLPaaadaahaaWcbeqaaiabikdaYaaaaOGaay5waiaaw2faamaaCaaaleqabaGaeGimaaJaeiOla4IaeGynauJae8NSdigaaaaaaaaaaa@8E82@

This cannot be solved for *x *using standard analytical solution techniques and therefore a simple numerical integration scheme was implemented using an explicit Runge-Kutta formula of 4^th^/5^th ^order. Both the analytical solution and the numerical solutions are plotted in Fig. 5, showing excellent agreement.

#### Flow at a bifurcation

Following the analysis of Smith et al [18], a bifurcation in the arterial network is approximated using three short elastic tubes which are short enough to assume a constant velocity along them and zero losses due to fluid viscosity. No fluid is assumed to be stored within the junction. The grid points associated with each vessel segment are shown in Fig. 6.

Equations (1) and (5) can be manipulated (for R ≠ 0) to obtain the following expression:

∂p∂t+12πR∂F∂xdpdR=0
 MathType@MTEF@5@5@+=feaafiart1ev1aaatCvAUfKttLearuWrP9MDH5MBPbIqV92AaeXatLxBI9gBaebbnrfifHhDYfgasaacH8akY=wiFfYdH8Gipec8Eeeu0xXdbba9frFj0=OqFfea0dXdd9vqai=hGuQ8kuc9pgc9s8qqaq=dirpe0xb9q8qiLsFr0=vr0=vr0dc8meaabaqaciaacaGaaeqabaqabeGadaaakeaadaWcaaqaaiabgkGi2kabdchaWbqaaiabgkGi2kabdsha0baacqGHRaWkdaWcaaqaaiabigdaXaqaaiabikdaYGGaciab=b8aWjabdkfasbaadaWcaaqaaiabgkGi2kabdAeagbqaaiabgkGi2kabdIha4baadaWcaaqaaiabdsgaKjabdchaWbqaaiabdsgaKjabdkfasbaacqGH9aqpcqaIWaamaaa@44CD@

Applying the principle of conservation of momentum for tube *a *yields:

πRa2(pa−po)=∂(ρlaπRa2Va)∂t
 MathType@MTEF@5@5@+=feaafiart1ev1aaatCvAUfKttLearuWrP9MDH5MBPbIqV92AaeXatLxBI9gBaebbnrfifHhDYfgasaacH8akY=wiFfYdH8Gipec8Eeeu0xXdbba9frFj0=OqFfea0dXdd9vqai=hGuQ8kuc9pgc9s8qqaq=dirpe0xb9q8qiLsFr0=vr0=vr0dc8meaabaqaciaacaGaaeqabaqabeGadaaakeaaiiGacqWFapaCcqWGsbGudaqhaaWcbaGaemyyaegabaGaeGOmaidaaOGaeiikaGIaemiCaa3aaSbaaSqaaiabdggaHbqabaGccqGHsislcqWGWbaCdaWgaaWcbaGaem4Ba8gabeaakiabcMcaPiabg2da9maalaaabaGaeyOaIyRaeiikaGIae8xWdiNaemiBaW2aaSbaaSqaaiabdggaHbqabaGccqWFapaCcqWGsbGudaqhaaWcbaGaemyyaegabaGaeGOmaidaaOGaemOvay1aaSbaaSqaaiabdggaHbqabaGccqGGPaqkaeaacqGHciITcqWG0baDaaaaaa@4E51@

Expressions similar to (13) and (14) can be written for tubes *b *and *c*. Expanding these equations using a central difference representation about (k+1/2) time step and further manipulation (refer to [18] for derivation details) of the resulting difference equations give:

pa1k+1−pb1k+1−2Δt(LaFa1k+1+LbFb1k+1)=−2Δt(LaFa1k+LbFb1k)+pbk−pak
 MathType@MTEF@5@5@+=feaafiart1ev1aaatCvAUfKttLearuWrP9MDH5MBPbIqV92AaeXatLxBI9gBaebbnrfifHhDYfgasaacH8akY=wiFfYdH8Gipec8Eeeu0xXdbba9frFj0=OqFfea0dXdd9vqai=hGuQ8kuc9pgc9s8qqaq=dirpe0xb9q8qiLsFr0=vr0=vr0dc8meaabaqaciaacaGaaeqabaqabeGadaaakeaacqWGWbaCdaqhaaWcbaGaemyyaeMaeGymaedabaGaem4AaSMaey4kaSIaeGymaedaaOGaeyOeI0IaemiCaa3aa0baaSqaaiabdkgaIjabigdaXaqaaiabdUgaRjabgUcaRiabigdaXaaakiabgkHiTmaalaaabaGaeGOmaidabaGaeuiLdqKaemiDaqhaamaabmaabaGaemitaW0aaSbaaSqaaiabdggaHbqabaGccqWGgbGrdaqhaaWcbaGaemyyaeMaeGymaedabaGaem4AaSMaey4kaSIaeGymaedaaOGaey4kaSIaemitaW0aaSbaaSqaaiabdkgaIbqabaGccqWGgbGrdaqhaaWcbaGaemOyaiMaeGymaedabaGaem4AaSMaey4kaSIaeGymaedaaaGccaGLOaGaayzkaaGaeyypa0JaeyOeI0YaaSaaaeaacqaIYaGmaeaacqqHuoarcqWG0baDaaWaaeWaaeaacqWGmbatdaWgaaWcbaGaemyyaegabeaakiabdAeagnaaDaaaleaacqWGHbqycqaIXaqmaeaacqWGRbWAaaGccqGHRaWkcqWGmbatdaWgaaWcbaGaemOyaigabeaakiabdAeagnaaDaaaleaacqWGIbGycqaIXaqmaeaacqWGRbWAaaaakiaawIcacaGLPaaacqGHRaWkcqWGWbaCdaqhaaWcbaGaemOyaigabaGaem4AaSgaaOGaeyOeI0IaemiCaa3aa0baaSqaaiabdggaHbqaaiabdUgaRbaaaaa@7742@

and

pa1k+1−pc1k+1−2Δt(LaFa1k+1+LcFc1k+1)=−2Δt(LaFa1k+LcFc1k)+pck−pak
 MathType@MTEF@5@5@+=feaafiart1ev1aaatCvAUfKttLearuWrP9MDH5MBPbIqV92AaeXatLxBI9gBaebbnrfifHhDYfgasaacH8akY=wiFfYdH8Gipec8Eeeu0xXdbba9frFj0=OqFfea0dXdd9vqai=hGuQ8kuc9pgc9s8qqaq=dirpe0xb9q8qiLsFr0=vr0=vr0dc8meaabaqaciaacaGaaeqabaqabeGadaaakeaacqWGWbaCdaqhaaWcbaGaemyyaeMaeGymaedabaGaem4AaSMaey4kaSIaeGymaedaaOGaeyOeI0IaemiCaa3aa0baaSqaaiabdogaJjabigdaXaqaaiabdUgaRjabgUcaRiabigdaXaaakiabgkHiTmaalaaabaGaeGOmaidabaGaeuiLdqKaemiDaqhaamaabmaabaGaemitaW0aaSbaaSqaaiabdggaHbqabaGccqWGgbGrdaqhaaWcbaGaemyyaeMaeGymaedabaGaem4AaSMaey4kaSIaeGymaedaaOGaey4kaSIaemitaW0aaSbaaSqaaiabdogaJbqabaGccqWGgbGrdaqhaaWcbaGaem4yamMaeGymaedabaGaem4AaSMaey4kaSIaeGymaedaaaGccaGLOaGaayzkaaGaeyypa0JaeyOeI0YaaSaaaeaacqaIYaGmaeaacqqHuoarcqWG0baDaaWaaeWaaeaacqWGmbatdaWgaaWcbaGaemyyaegabeaakiabdAeagnaaDaaaleaacqWGHbqycqaIXaqmaeaacqWGRbWAaaGccqGHRaWkcqWGmbatdaWgaaWcbaGaem4yamgabeaakiabdAeagnaaDaaaleaacqWGJbWycqaIXaqmaeaacqWGRbWAaaaakiaawIcacaGLPaaacqGHRaWkcqWGWbaCdaqhaaWcbaGaem4yamgabaGaem4AaSgaaOGaeyOeI0IaemiCaa3aa0baaSqaaiabdggaHbqaaiabdUgaRbaaaaa@774E@

Equations (15) and (16) along with conservation of mass given by:

Fa1k+1−Fb1k+1−Fc1k+1=0
 MathType@MTEF@5@5@+=feaafiart1ev1aaatCvAUfKttLearuWrP9MDH5MBPbIqV92AaeXatLxBI9gBaebbnrfifHhDYfgasaacH8akY=wiFfYdH8Gipec8Eeeu0xXdbba9frFj0=OqFfea0dXdd9vqai=hGuQ8kuc9pgc9s8qqaq=dirpe0xb9q8qiLsFr0=vr0=vr0dc8meaabaqaciaacaGaaeqabaqabeGadaaakeaacqWGgbGrdaqhaaWcbaGaemyyaeMaeGymaedabaGaem4AaSMaey4kaSIaeGymaedaaOGaeyOeI0IaemOray0aa0baaSqaaiabdkgaIjabigdaXaqaaiabdUgaRjabgUcaRiabigdaXaaakiabgkHiTiabdAeagnaaDaaaleaacqWGJbWycqaIXaqmaeaacqWGRbWAcqGHRaWkcqaIXaqmaaGccqGH9aqpcqaIWaamaaa@44A8@

form a system of three nonlinear equations which are then solved using a Newton-Rhapson iterative scheme which attempts to simultaneously satisfy Equations (15) – (17).

Flow was simulated for the aorto-iliac bifurcation and the resulting numerical values satisfied the conservation of mass constraint with a 0.02% error (see Table 2).

**Table 2:** Conservation of mass at the aorto-iliac bifurcation during steady state

		Steady state
Parent vessel *F*_*p*_	R (mm)	11.12
	V (mm/s)	870.32
	F (mm^3^/s)	338094.74
Daughter Vessel 1 *F*_*d*1_	R (mm)	8.85
	V (mm/s)	727.05
	F (mm^3^/s)	178896.03
Daughter Vessel 2 *F*_*d*2_	R (mm)	8.26
	V (mm/s)	742.39
	F (mm^3^/s)	159126.34
% Error =Fp−(Fd1+Fd2)Fp×100% MathType@MTEF@5@5@+=feaafiart1ev1aaatCvAUfKttLearuWrP9MDH5MBPbIqV92AaeXatLxBI9gBaebbnrfifHhDYfgasaacH8akY=wiFfYdH8Gipec8Eeeu0xXdbba9frFj0=OqFfea0dXdd9vqai=hGuQ8kuc9pgc9s8qqaq=dirpe0xb9q8qiLsFr0=vr0=vr0dc8meaabaqaciaacaGaaeqabaqabeGadaaakeaacqqGLaqjcqqGGaaicqqGfbqrcqqGYbGCcqqGYbGCcqqGVbWBcqqGYbGCcqqGGaaicqqG9aqpdaWcaaqaaiabdAeagnaaBaaaleaacqWGWbaCaeqaaOGaeyOeI0IaeiikaGIaemOray0aaSbaaSqaaiabdsgaKjabigdaXaqabaGccqGHRaWkcqWGgbGrdaWgaaWcbaGaemizaqMaeGOmaidabeaakiabcMcaPaqaaiabdAeagnaaBaaaleaacqWGWbaCaeqaaaaakiabgEna0kabigdaXiabicdaWiabicdaWiabcwcaLaaa@4C8B@		0.02%

#### Numerical stability

The two characteristic paths along which information propagates in *(x,t) *space for the governing equations are given by [18]:

dxdt=αV±[α(α−1)V2+βG0Rβ2ρR0β]0.5,
 MathType@MTEF@5@5@+=feaafiart1ev1aaatCvAUfKttLearuWrP9MDH5MBPbIqV92AaeXatLxBI9gBaebbnrfifHhDYfgasaacH8akY=wiFfYdH8Gipec8Eeeu0xXdbba9frFj0=OqFfea0dXdd9vqai=hGuQ8kuc9pgc9s8qqaq=dirpe0xb9q8qiLsFr0=vr0=vr0dc8meaabaqaciaacaGaaeqabaqabeGadaaakeaadaWcaaqaaiabdsgaKjabdIha4bqaaiabdsgaKjabdsha0baacqGH9aqpiiGacqWFXoqycqWGwbGvcqGHXcqSdaWadaqaaiab=f7aHjabcIcaOiab=f7aHjabgkHiTiabigdaXiabcMcaPiabdAfawnaaCaaaleqabaGaeGOmaidaaOGaey4kaSYaaSaaaeaacqWFYoGycqWGhbWrdaWgaaWcbaGaeGimaadabeaakiabdkfasnaaCaaaleqabaGae8NSdigaaaGcbaGaeGOmaiJae8xWdiNaemOuai1aa0baaSqaaiabicdaWaqaaiab=j7aIbaaaaaakiaawUfacaGLDbaadaahaaWcbeqaaiabicdaWiabc6caUiabiwda1aaakiabcYcaSaaa@556F@

or

dxdt=αV±[α(α−1)V2+c2]0.5
 MathType@MTEF@5@5@+=feaafiart1ev1aaatCvAUfKttLearuWrP9MDH5MBPbIqV92AaeXatLxBI9gBaebbnrfifHhDYfgasaacH8akY=wiFfYdH8Gipec8Eeeu0xXdbba9frFj0=OqFfea0dXdd9vqai=hGuQ8kuc9pgc9s8qqaq=dirpe0xb9q8qiLsFr0=vr0=vr0dc8meaabaqaciaacaGaaeqabaqabeGadaaakeaadaWcaaqaaiabdsgaKjabdIha4bqaaiabdsgaKjabdsha0baacqGH9aqpiiGacqWFXoqycqWGwbGvcqGHXcqSdaWadaqaaiab=f7aHjabcIcaOiab=f7aHjabgkHiTiabigdaXiabcMcaPiabdAfawnaaCaaaleqabaGaeGOmaidaaOGaey4kaSIaem4yam2aaWbaaSqabeaacqaIYaGmaaaakiaawUfacaGLDbaadaahaaWcbeqaaiabicdaWiabc6caUiabiwda1aaaaaa@497B@

where *c *is the wave speed at zero mean flow.

For the numerical scheme to be stable, the numerical velocity (ΔxΔt
 MathType@MTEF@5@5@+=feaafiart1ev1aaatCvAUfKttLearuWrP9MDH5MBPbIqV92AaeXatLxBI9gBaebbnrfifHhDYfgasaacH8akY=wiFfYdH8Gipec8Eeeu0xXdbba9frFj0=OqFfea0dXdd9vqai=hGuQ8kuc9pgc9s8qqaq=dirpe0xb9q8qiLsFr0=vr0=vr0dc8meaabaqaciaacaGaaeqabaqabeGadaaakeaadaWcaaqaaiabfs5aejabdIha4bqaaiabfs5aejabdsha0baaaaa@3272@) of the finite difference scheme has to be greater than the wave speed of the equations, or else errors will be introduced which will ultimately grow and make the solutions unstable. That is:

ΔxΔt>|dxdt|
 MathType@MTEF@5@5@+=feaafiart1ev1aaatCvAUfKttLearuWrP9MDH5MBPbIqV92AaeXatLxBI9gBaebbnrfifHhDYfgasaacH8akY=wiFfYdH8Gipec8Eeeu0xXdbba9frFj0=OqFfea0dXdd9vqai=hGuQ8kuc9pgc9s8qqaq=dirpe0xb9q8qiLsFr0=vr0=vr0dc8meaabaqaciaacaGaaeqabaqabeGadaaakeaadaWcaaqaaiabfs5aejabdIha4bqaaiabfs5aejabdsha0baacqGH+aGpcqGG8baFdaWcaaqaaiabdsgaKjabdIha4bqaaiabdsgaKjabdsha0baacqGG8baFaaa@3C16@

Substituting (18) into (19) we get:

ΔxΔt>|αV±[α(α−1)V2+c2]0.5|
 MathType@MTEF@5@5@+=feaafiart1ev1aaatCvAUfKttLearuWrP9MDH5MBPbIqV92AaeXatLxBI9gBaebbnrfifHhDYfgasaacH8akY=wiFfYdH8Gipec8Eeeu0xXdbba9frFj0=OqFfea0dXdd9vqai=hGuQ8kuc9pgc9s8qqaq=dirpe0xb9q8qiLsFr0=vr0=vr0dc8meaabaqaciaacaGaaeqabaqabeGadaaakeaadaWcaaqaaiabfs5aejabdIha4bqaaiabfs5aejabdsha0baacqGH+aGpcqGG8baFiiGacqWFXoqycqWGwbGvcqGHXcqSdaWadaqaaiab=f7aHjabcIcaOiab=f7aHjabgkHiTiabigdaXiabcMcaPiabdAfawnaaCaaaleqabaGaeGOmaidaaOGaey4kaSIaem4yam2aaWbaaSqabeaacqaIYaGmaaaakiaawUfacaGLDbaadaahaaWcbeqaaiabicdaWiabc6caUiabiwda1aaakiabcYha8baa@4CB1@

Velocity of blood is seldom greater than 1 m/s while *c *is approximately 5 m/s [32]. Using an α value of 1.1 the stability criterion is approximately:

ΔxΔt>α|V|+c
 MathType@MTEF@5@5@+=feaafiart1ev1aaatCvAUfKttLearuWrP9MDH5MBPbIqV92AaeXatLxBI9gBaebbnrfifHhDYfgasaacH8akY=wiFfYdH8Gipec8Eeeu0xXdbba9frFj0=OqFfea0dXdd9vqai=hGuQ8kuc9pgc9s8qqaq=dirpe0xb9q8qiLsFr0=vr0=vr0dc8meaabaqaciaacaGaaeqabaqabeGadaaakeaadaWcaaqaaiabfs5aejabdIha4bqaaiabfs5aejabdsha0baacqGH+aGpiiGacqWFXoqycqGG8baFcqWGwbGvcqGG8baFcqGHRaWkcqWGJbWyaaa@3B86@

and this was the condition that was used when determining the maximum value of Δt for a given Δx.

## Results

Using the same parameter values as in the previous section and an initial condition of 10.2 kPa, the flow in the mesenteric arterial model shown in Fig. 3 was simulated using a periodic inlet boundary condition pressure pulse at the proximal abdominal aorta, varying between 10.2 kPa (77 mmHg) and 14.6 kPa (110 mmHg). This pressure profile (see Fig. 7) was based on data from [31] and represents the four distinct cardiac contraction phases-diastole, isovolumic contraction, ejection and isovolumic relaxation. The exit boundary condition was specified as a constant pressure of 11 kPa and we believed this was a reasonable value to hold the exit boundary condition at since at the sizes of the vessels we are interested in, we expect the exit pressures to be something between the minimum and the maximum of the input pressure pulse. Using our simulation software, it is possible to specify temporally varying exit boundary conditions as well, but any realistic, time-varying exit boundary conditions corresponding to the inlet pressure cannot be easily determined. A solution to this is to include the microcirculation and the venous network as well and to set the terminal venous pressure to a low value such as 0 kPa, similar to the work in [18], but it simply is not a possibility at this stage with the VH images where majority of the small vessels are either collapsed or simply not visible.

The boundary and initial conditions used in (1), (4) and (5) are summarized as follows:

pinlet(t)= Temporally varying pressure profile specified in Fig. 7poutlet=11 kPaRi0=Ri0Vi0=0
 MathType@MTEF@5@5@+=feaafiart1ev1aaatCvAUfKttLearuWrP9MDH5MBPbIqV92AaeXatLxBI9gBaebbnrfifHhDYfgasaacH8akY=wiFfYdH8Gipec8Eeeu0xXdbba9frFj0=OqFfea0dXdd9vqai=hGuQ8kuc9pgc9s8qqaq=dirpe0xb9q8qiLsFr0=vr0=vr0dc8meaabaqaciaacaGaaeqabaqabeGadaaakqaabeqaaiabdchaWnaaBaaaleaacqWGPbqAcqWGUbGBcqWGSbaBcqWGLbqzcqWG0baDaeqaaOGaeiikaGIaemiDaqNaeiykaKIaeyypa0JaeeiiaaIaeeivaqLaeeyzauMaeeyBa0MaeeiCaaNaee4Ba8MaeeOCaiNaeeyyaeMaeeiBaWMaeeiBaWMaeeyEaKNaeeiiaaIaeeODayNaeeyyaeMaeeOCaiNaeeyEaKNaeeyAaKMaeeOBa4Maee4zaCMaeeiiaaIaeeiCaaNaeeOCaiNaeeyzauMaee4CamNaee4CamNaeeyDauNaeeOCaiNaeeyzauMaeeiiaaIaeeiCaaNaeeOCaiNaee4Ba8MaeeOzayMaeeyAaKMaeeiBaWMaeeyzauMaeeiiaaIaee4CamNaeeiCaaNaeeyzauMaee4yamMaeeyAaKMaeeOzayMaeeyAaKMaeeyzauMaeeizaqMaeeiiaaIaeeyAaKMaeeOBa4MaeeiiaaIaeeOrayKaeeyAaKMaee4zaCMaeeOla4IaeeiiaaIaee4naCdabaGaemiCaa3aaSbaaSqaaiabd+gaVjabdwha1jabdsha0jabdYgaSjabdwgaLjabdsha0bqabaGccqGH9aqpcqaIXaqmcqaIXaqmcqqGGaaicqqGRbWAcqqGqbaucqqGHbqyaeaacqWGsbGudaqhaaWcbaGaemyAaKgabaGaeGimaadaaOGaeyypa0JaemOuai1aa0baaSqaaiabdMgaPbqaaiabicdaWaaaaOqaaiabdAfawnaaDaaaleaacqWGPbqAaeaacqaIWaamaaGccqGH9aqpcqaIWaamaaaa@9F9E@

**Figure 8:**
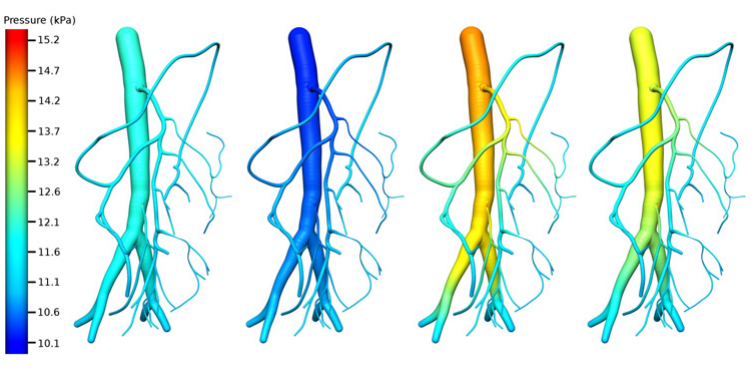
Pressure distribution of the 1D mesenteric artery network at selected times. (a) Pressure distribution during diastole (t = 0.55 s) (b); at the end of isovolumic contraction (t = 0.98 s); (c) at peak ejection (t = 1.31 s); (d) at peak isovolumic relaxation (t = 1.46 s).

**Figure 9:**
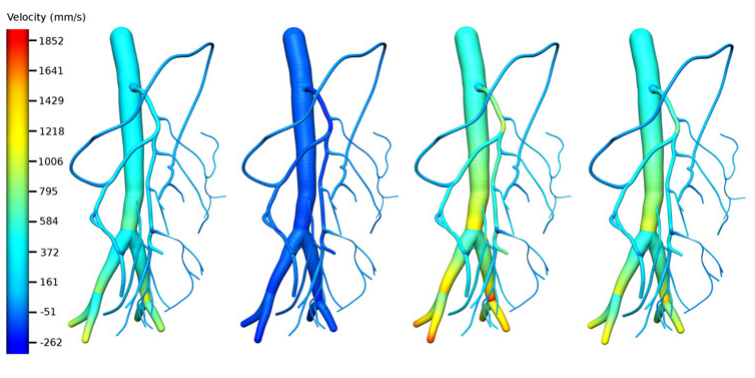
Velocity distribution of the 1D mesenteric artery network. (a) Velocity distribution during diastole (t = 0.55 s); (b) at the end of isovolumic contraction (t = 0.98 s); (c) at peak ejection (t = 1.31 s); (d) at peak isovolumic relaxation (t = 1.46 s).

The flow equations were solved for pressure, radius and velocity, but only pressure and velocity results are presented herein as the radii changes over time were less than ± 5% of initial radius. The observed pressure and velocity profiles of the 1-D mescenteric artery network at four distinct times are shown in Figs. 8 and 9.

The above profiles were generated using a time resolution Δt of 0.2 ms, and a spatial resolution Δx of 1.3 mm which guaranteed at least 10 grid points for each vessel segment. This value of Δt was chosen based on the stability criterion (Section II(5)) and was the maximum theoretical time resolution that could be used with the smallest spatial discritization. Pushing Δt to its maximum theoretical limit helps in reducing simulation time and computational effort.

Our results indicate that the simulation is numerically stable and is conserving mass and momentum. The profiles also show that continuity of pressure is maintained across all bifurcations points as expected. It is interesting to note that there is almost always an increase in the pressure along the vessel proximal to a bifurcation, and this is most evident during peak ejection (see Fig. 8(c)) where the pressure gradient in the network is the steepest. This is because as vessels bifurcate, giving rise to daughter vessels whose area sum is greater than the area of the parent (i.e., an increased vessel wall surface area to volume ratio), the pressure drops which results in an increased friction factor. Note that even though the total cross-sectional area has increased, the cross-sectional area for each daughter vessel has decreased resulting in a decreased velocity and pressure. Our geometry is consistent with the findings of [35] where an average area increase of 1.26 was reported at a bifurcation. No one has ever observed an increase in area as large as a factor as 2 at any bifurcation [31] and it is deemed that the vascular resistance measured at any branch will always be higher than that of the parent since vascular resistance is largely determined by the radius of the vessels. It should be noted that some literature have reported that arterial bifurcations are normally fractal in nature and follow Murray's law with an exponential factor usually ranging from 2.3–2.7 [36], but it should also be noted that in these theoretical studies the vessels are usually modeled as straight, rigid cylindrical tubes, often with identical daughter vessels.

Similar to the pressure profiles, a drop in velocity can be observed at each bifurcation as the area sum of daughter vessels is greater than the area of the parent. However, unlike pressure, velocity is not continuous across a bifurcation. During the diastole phase, arterial velocity decreases as the pressure boundary condition at the descending aorta drops (hence smaller pressure gradients across the network), while arteriole velocity rises as flow begins to be transmitted through vessels with smaller radii. Similar to the observations made by Chakravarty et al [7] and Pedersen et al [37], a small backflow can be observed during this phase.

It is interesting to note that the maximum abdominal aortic velocity our simulations give, around 600 mm/s (see Fig. 9(c)), compares well with the 59 cm/s measured *in vivo *by Pedersen et al [37]. Their minimum abdominal aortic velocity of approximately -10 cm/s also matches extremely well with our simulated minimum abdominal aortic velocity (see Fig. 9(b)). With the onset of ejection, the rapid rise in aortic pressure begins to dominate velocities. The rising pressure produces a drop in radii in the low pressure, arteriole network, resulting in an increase in velocity as blood is squeezed out of these vessels.

**Figure 10:**
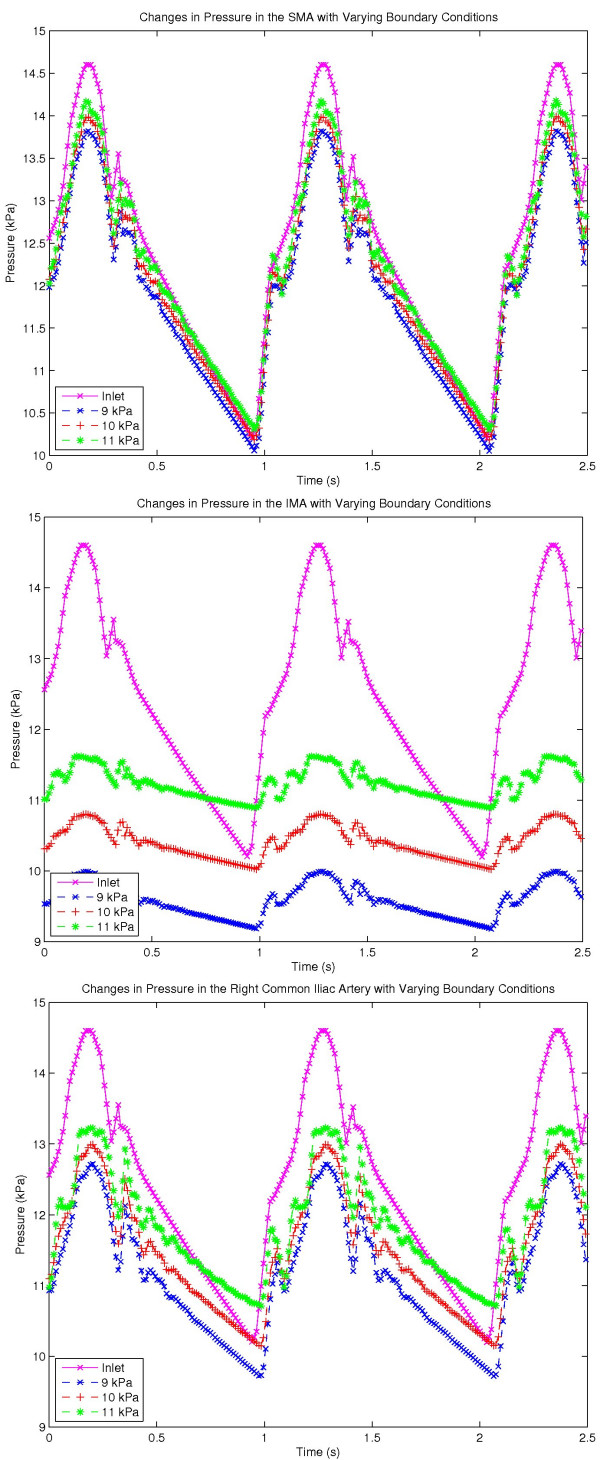
Graphs showing the variation in pressure at various locations in the 1D mesenteric arterial tree, (a) SMA (b) IMA (c) Right common iliac artery. The pressure keys (9 kPa, 10 kPa and 11 kPa) in these graphs indicate the exit pressure boundary condition.

We also examined the sensitivity of the constant-terminal-pressure boundary condition we have used by running the same simulations as those discussed previously, but holding the terminal pressures at different values. Temporal variations in pressure at chosen locations of the arterial tree were observed, and Figs 10(a–c) show the pressure variation in the SMA, IMA and the right common iliac artery with these varying exit pressures. The abdominal aortic input pressure waveform (similar to Fig. 7) is also shown for comparison purposes.

Fig. 10(a) shows the minimum sensitivity to the variations in exit pressure and this is because the SMA is the closest to the input of the above three arteries and the high velocities in the abdominal aorta propagate the input pressure pulse to the SMA with minimal delay. Fig 10(b) is towards an exit point and the maximum change in pressure observed, compared to the terminal pressure is only about 0.7 kPa irrespective of the boundary condition. The amplitude of the input pressure pulse has gradually decreased towards the common iliac arteries (Fig. 10(c)) although the same waveform can be observed, but with a slight delay due to propagation delays. Also, it is interesting to note that the minimum propagation delays are observed when the pressure gradient in the arterial tree is maximum (that is when the terminal pressures are maintained at 9 kPa in the above simulations), which is the result of increased velocities due to increasing pressure gradients.

## Discussion

We have presented an anatomically realistic computational model of the major mesenteric arteries (constituting mainly of the abdominal aorta, SMA, IMA, common iliac arteries and the middle colic artery) and used it to simulate normal blood flow. This has been successfully achieved by digitizing the male VH dataset and creating a finite element mesh on which a one dimensional numerical computational analysis has been performed. In addition, we have presented an analytical scheme which can be used to determine vessel radius at a given location for a tapering vessel with a steady pressure gradient.

Early diagnosis of mesenteric ischemia is vital and we believe the framework presented herein can be used to achieve this ultimate goal. Narrowing of vessels is not uncommon and with the many redundant systems (loops) in the mesenteric arterial network, narrowed vessels do not necessarily indicate mesenteric ischemia – therefore perfusion profiles need to be looked at to get an indication on the bowel movement. The unstressed radius (R_0_) of the mesenteric network of a suspected patient can be determined using an MRI (or some other screening technique) and using the techniques presented here, the radii and velocities at each solution point can be determined. Using these time varying radii and velocities, perfusion profiles can be developed which can then be examined by a medical expert for early diagnostic purposes.

As pointed out by many authors, including Olufsen et al [9], Wilson et al [21] and Quarteroni [5], numerical simulation of the vascular system can offer great insight into the physiological behavior of the hemodynamics of the human cardiovascular system. Patient specific geometric models of the arteries can be created using various imaging modalities such as MRI, CT, X-Ray, MRA and ultrasound which can then be used to perform physiologically realistic numerical simulations. Such a tool can be very useful in a clinical environment, especially in the diagnosis, understanding and monitoring the progression of cardiovascular diseases.

Our simulations are based on some fundamental assumptions (such as Newtonian blood, laminar flow, zero radial velocity and so on as described in Section II(4)) which are broadly accepted in published literature for one dimensional models. Apart from these flow assumptions, an important assumption made is the pressure-radius relationship where a purely elastic vessel wall is assumed. Such an assumption was considered acceptable by Saito et al [33] in their studies as the effects of including a viscoelastic model were fairly minimal, although such viscoelastic walls may have some effect [38]. Also, the vessels are assumed to be in a maximally dilated state where any effects of the vascular smooth muscle are not taken into account.

In our current work, arterial blood flow was simulated starting from a single vessel segment, which was then extended to a bifurcation using the conservation of mass and momentum equations and a constitutive pressure-radius relationship. The technique was then applied to the computational mesenteric arterial network that was created and the results in the abdominal aortic region show a realistic pressure and velocity distribution when compared with the in vivo measurements made by [37]. However, this is by no means an indication that the current model is without its own set of drawbacks and limitations. One of the main drawbacks of the current model is that it is based on images from the male VH dataset which represent the geometry of a deceased person, and therefore some of the vessels have either collapsed or are difficult to identify. Also, the model is based on some material parameters (such as G_0 _and β which control the compliance of the vessels) which have not been measured in a clinical setting. These parameters can be expected to vary throughout the arterial tree, although a constant value has been assumed for the entire network in this work mainly due to the lack of detailed knowledge. However, varying material parameter values can easily be accommodated into the existing framework.

Another major drawback with the current model is its inability to create the capillary and venous networks. It is possible to assume identical geometries for the arterial and venous networks and run similar simulations as in [18] by using a lumped parameter model for the microcirculation in the capillary network, but this may not be a valid assumption since the arterial and venous networks have considerably different vascular geometries *in vivo*. Despite the rapid developments in imaging modalities, the complexity and the practical limitations associated with resolving the microvasculature are very likely to make lumped parameter models the preferred method for microcirculation simulations even in the foreseeable future.

To remedy the issues encountered due to indistinct vessel boundaries in the male VH images, we now intend to develop a new model based on MRI images of a healthy volunteer, which should lead to a better anatomical description of the network of a living human. Modeling mesenteric arterial blood flow will hopefully increase our understanding of blood supply to the intestine and help us develop a generic model of the mesenteric arteries (to some extent similar to the work that has been done on the human heart [19]) with a range of acceptable vessel radii values and geometries, which patient specific models can be compared against.

Ultimately, we intend to use our model to simulate ischemic conditions as occur in those suffering from mesenteric ischemia. This can also be easily accommodated into the existing framework as R_0_, the most noticeable change during a stenotic condition is already a model parameter. Future work will involve using that complete model to simulate compromised blood flow as seen during mesenteric ischemia. This will allow us to assess the degree to which different levels of flow restrictions in different vessels affect different regions of the network.

## Conclusion

A computational model of the major arteries of the gastrointestinal system has been developed based on VH images, which shows anatomically and physiologically realistic geometries. Blood flow during normal conditions has been simulated using this computational model and results indicate that numerical flow modeling on a complex system such as the mesenteric network is feasible and would yield realistic flow profiles.

## Competing interests

The author(s) declare that they have no competing interests.

## Authors' contributions

Author TDSM created the final computational model based on VH images, performed the various simulations required, analyzed the results and drafted the manuscript. LKC and AJP were actively involved in the supervision and development of the research and revised the manuscript. All authors read and approved the final version of the manuscript.
